# Quality of Fixed Dental Prostheses and Patient Satisfaction in a Sample From Saudi Arabia

**DOI:** 10.7759/cureus.51063

**Published:** 2023-12-25

**Authors:** Ahmed H Albaqawi, Mohammad D Aljanakh, Bayan N Alshammari, Modhi A Alshammari, Rawan H Alshammari, Ghadeer D Alshammari, Bodor Z Alshammari, Rawan A AlShammari, Ruqayyah F Alturki, Ahmed A Madfa

**Affiliations:** 1 Restorative Dental Science, College of Dentistry, University of Ha’il, Ha’il, SAU; 2 Restorative Dental Science, College of Dentistry, University of Ha'il, Ha’il, SAU; 3 Dentistry, College of Dentistry, University of Ha'il, Ha'il, SAU; 4 Dentistry, College of Dentistry, University of Ha'il, Ha’il, SAU; 5 Dentistry, College of Dentistry, University of Ha'il, Haˈil, SAU; 6 Restorative Dental Science, College of Dentistry, University of Ha'il, Ha'il, SAU

**Keywords:** patient satisfaction, crown, biological and mechanical complication, fixed dental prosthesis, single crown

## Abstract

Background: The objective of the study was to retrospectively assess the clinical performance of dental prostheses, single crowns, and three-unit bridges to identify clinical biological and mechanical complications in the Ha'il province of Saudi Arabia.

Methods: The study was conducted between March 2021 to October 2021 and included 421 patients who underwent crown and tooth-supported fixed partial denture (FPD) procedures at the Dental Polyclinics Center in the Ha'il region of Saudi Arabia from 2010-2020. The planned sampling approach was applied. Patients who underwent crown and FPD placements at the dental center within the scheduled period were identified through clinical assessments. The inclusion criteria were met by patients with three-unit FPDs and a single crown containing a natural contralateral tooth or teeth. A total of six general dentists conducted clinical exams. Patient satisfaction and technical and biological issues were the evaluation criteria for crowns and FPDs. The frequency and percentage of the various characteristics employed in the current investigation were ascertained using cross-tabulation. The Chi-square test was employed to assess the associations between categorical variables, with p-values ≤ 0.05 considered significant.

Results: Marginal integrity was recorded in 332 participants (78.9%), which was satisfactory for the majority of the restoration. The acceptance morphology was present in 252 (59.9%) restorations. The highest rate of the restorations 274 (66.2%) had satisfactory color. In 86 cases (20.4%), there was visible periodontal depth of at least 5 mm. Three hundred and six (72.7%) of the fixed restorations had gingival bleeding connected to them, and 96 (22.8%) teeth had periapical lesions. A total of 311 patients (73.9%) reported they were satisfied with their fixed restorations.

Conclusions: The technical and biological complications noted in the current study were not higher compared with other studies of crowns and fixed dental prostheses. The majority of patients were satisfied with the restoration.

## Introduction

Fixed dental prostheses (FDPs) are artificial teeth that serve as a permanent replacement for lost teeth by being affixed to the remaining teeth. One or more lost or missing teeth can be functionally restored with the help of the commonly utilized single crown and FDP (bridge) treatment approach. There will always be conditions in which fixed prosthetics are necessary to replace lost teeth [1]. Due to the development of full-ceramic fixed partial denture (FPD) materials and their availability for clinical usage, all-ceramic FPDs are now often utilized in clinical dentistry. The success rate for anterior ceramic crowns and veneers is very high. Olley et al. followed up with patients for 50 years, during which all the ceramic crowns and veneers survived [2]. The only failures occurred with metal-ceramic crowns. On the other hand, it has been noted that one of the main reasons these restorations fail is fractures of the posterior all-ceramic FPDs [3].

Fixed restorations continue to be essential elements of prosthodontic restoration practice overall, especially in countries that are developing, despite the growing popularity of dental implants [4]. On the other hand, a ceramic crown requires several intricate steps that are completed in phases. Consequently, there's a good chance that the material will become brittle due to the formation of voids. Material characteristics may be improved by substituting automated processes for manual ones, as in computer-aided design/computer-aided manufacturing (CAD/CAM) systems. The likelihood of clinical success could be increased by the automated production processes by improving the homogeneity of ceramic restorations.

For biological investment tissue restoration and health maintenance to be successful and durable, diligent case selection, comprehensive diagnosis, precise preparation, and skilled prosthesis manufacturing are necessary [5,6]. On the other side, an improperly maintained prosthesis has a higher chance of failing early and catastrophic damage to the teeth and tissues underneath [7].

Prevalence of dental caries was the most frequent complication with FDP, as reported by Goodacre et al. [6]. They found that 11% of abutments had teeth that needed a root canal after a prosthesis, and 18% of abutments had caries. Complications (4% of prostheses) included aesthetics (6%), periodontal disease (4%), tetracycline (4% of prostheses), and loss of retention (7% of prostheses). Porcelain fracture (3%), restoration loss (2%), necrotic pulp (3%), periodontal disease (0.6%), and caries (2%), were the most frequent issues related to fixed prostheses. The most frequent issue resulting from post and core complications was post loosening (5%) which was followed by root fractures (3%), periodontal disease (2%), and denatl caries (2%) [6].

Clinical success for patients receiving restorations depends significantly on patient satisfaction. A direct assessment of patients' opinions and sentiments regarding several aspects of prosthodontic rehabilitation is made possible by the examination of satisfaction results. Patient satisfaction with ceramic treatments is influenced by improvements in function, comfort, and aesthetics as well as oral health [8].

Treatment involving FPDs and crowns requires a particular set of abilities. It is among the most often used dental treatment modalities. Previous studies examined and documented patient satisfaction with all-ceramic restorations about treatment satisfaction and oral hygiene [2,8]. Nevertheless, there is a lack of published research on the factors influencing patient satisfaction with ceramic restorations and their clinical results in a subset of Saudi patients. Therefore, evaluating the success and longevity of manufactured repairs, along with the origins and nature of issues and malfunctions related to these prostheses, is essential. Gaining knowledge of these factors can help dentists create the best possible treatment plan, set reasonable expectations for their patients, and create a maintenance schedule that works for patients with fixed prostheses [6,9].

To the best of our knowledge, no research has been done in the Ha'il region on patient satisfaction or the caliber of treatment done on crowns and fixed partial dentures. To ascertain the prevalence of issues related to crowns and fixed restorations in a Saudi subpopulation using clinical and radiographic indicators, this study aimed to evaluate patient satisfaction and the work quality of crowns and FPDs. 

This article was previously posted to the medRxiv preprint server on May 25, 2022.

## Materials and methods

This study was approved by the Ethical Committee of the University of Hail College of Dentistry (approval number: H-2021-63, dated May 15, 2020). It was conducted in the Ha'il Dental Polyclinics Center. A list of patients who had crowns and FPDs fixed between 2010 and 2020 was obtained through clinical evaluation. Data collection started at the beginning of 2021. A total of 421 patients who met the requirements for this study's inclusion were located.

Inclusion and exclusion criteria

Patients with at least three units of FPDs or a single crown with the presence of natural contra-lateral tooth/teeth were included. All FPDs performed and inserted by general dental practitioners were also included. Patients with FPDs of more than three units, fixed partial with the absence of natural contra-lateral tooth/teeth, and previously repaired fixed partial dentures were excluded. 

Data collection and evaluation

To determine which patients at the dental center received crowns and FPDs within the scheduled time frame, clinical and radiographic evaluations were conducted. All patients who responded and showed up for evaluation were included in the research study when the consenting procedures were completed.

Six general dentists were calibrated and trained by a prosthodontist for one week before starting the study. Subsequently, the collected data were evaluated by two examiners for each patient to verify the interreliabilities. Table 1 displays the evaluation criteria for the crowns and FPDs using the California Dental Association (CDA) guidelines for the assessment of clinical quality and professional performance.

**Table 1:** Parameters recorded in this study

Definition	Criteria	Parameters
Technical criteria or consideration		
Marginal integrity	Acceptable	Detectable slight marginal discrepancy; repair is unnecessary. Discoloration between the crown and the tooth.
	Unacceptable	Restoration is mobile, lost, or fractured, or caries contiguous with the margin or restoration or tooth structure fractured.
Morphology	Acceptable	Continuous contour with an existing anatomical form of the adjacent and contralateral teeth with minor deviations.
	Retrievable	Deviations that create discomfort. Can be adjusted.
	Unacceptable	Restoration causes pain in the tooth or adjacent tissue.
Color	Acceptable	Slight mismatch between shade of the restoration and adjacent teeth, within normal range of tooth color, shade and/or translucency.
	Unacceptable	Mismatch between restoration and adjacent teeth outside normal range of color, shade and/or translucency
Biological criteria or consideration		
Periodontal depth	Less than 5 mm	
	Equal or more than 5 mm	
Gingiva bleeding	Bleeding on probing	
	No bleeding on probing	
Periapical lesion	Yes	If the widening of the apical part of the periodontal ligament or if the periapical radiolucency in connection with the apical part of the tooth exceeds at least two times the width of the lateral part of the periodontal ligament, such teeth were categorized as having obvious periapical radiolucency.
	No	If the periodontal ligament was intact with no signs of periapical disease.
Ceramic surface	Yes	Chipping of ceramic impairing esthetics and function or exposing tooth structure; intraceramic fissures detectable with the explorer.
	No	Smooth surface (shiny after air-drying).
Dental carious	Yes	Caries is evident contiguous with the margin of the restoration
	No	No evidence of caries contiguous with the margin of the restoration.
Other parameters		
Satisfaction	Yes	
	No	
Materials	PFM	
	Zirconia	
	Other	
Types	Crown	
	3 units bridge	
Location	Anterior	
	Posterior	

Data analysis

For statistical studies, IBM SPSS Statistics for Windows, Version 25.0 (Released 2017; IBM Corp., Armonk, New York, United States) was utilized with a significance level of 0.05 to determine statistical significance. The frequency and percentage of the various characteristics employed in the current investigation are ascertained using cross-tabulation.

## Results

Table 2 displays the technical findings. Marginal integrity was satisfactory for the majority of the restorations (n=332; 78.9%). Only 89 (21.1%) cases had non-acceptable marginal integrity. Only 43 (10.2%) of the restorations had unacceptable morphology, while the majority (n=252; 59.9%) had acceptable shape. In 126 cases (29.9%), retrievable morphology was detected. Of all the restorations, 274 (66.2%) had satisfactory color, while 140 (33.8%) had unsatisfactory color and the remaining seven samples were metallic crowns and, therefore, were not considered in the color finding.

**Table 2:** Technical findings recorded in the present study

Parameter	Measurement	n (%)
Marginal integrity	Acceptable	332 (78.9)
	Non-acceptable	89 (21.1)
Morphology	Acceptable	252 (59.9)
	Retrievable	126 (29.9)
	Non-acceptable	43 (10.2)
Color	Acceptable	274 (66.2)
	Non-acceptable	140 (33.8)

Biological findings as a result of restorations are shown in Table 3. Visible periodontal depths equal to or more than 5 mm were found in 86 (20.4%). Most of the fixed restorations were associated with gingival bleeding (n=306; 72.7%), while periapical lesion was found on 96 (22.8) teeth. Dental caries were found in 42 (10.0%) of the restored teeth.

**Table 3:** Biological findings recorded in the present study

Parameter	Measurement	n (%)
Periodontal depth	Less than 5 mm	335 (79.6)
	Equal or more than 5 mm	86 (20.4)
Gingiva bleeding	Bleeding on probing	306 (72.7)
	No bleeding on probing	115 (27.3)
Periapical lesion	Yes	96 (22.8)
	No	325 (77.2)
Loosening of FPD	Yes	29 (6.9)
	No	392 (93.1)
Dental carious	Yes	42 (10.0)
	No	379 (90.0)

Table 4 shows patient satisfaction together with other fixed restoration parameters. A total of 311 patients (73.9%) agreed they were satisfied with their fixed restorations. The restorations had left the remaining patients dissatisfied. Porcelain-fused-to-metal (PFM) accounted for the majority of the restorations studied in this study (n=242; 57.5%), whereas 165 (39.2%) had zirconia crowns. There were just 14 (3.4%) other restorations. Of the patients that were investigated, 307 (72.9%) had crown restorations recorded, whereas 114 (27.1%) had a three-unit bridge presented. The posterior 279 teeth had the greatest number of fixed restorations (66.3%). The majority of FDPs had a duration of 5-10 years (31.8%).

**Table 4:** Other parameters reported in the present study

Parameter	Measurement	n (%)
Satisfaction	Yes	311 (73.9)
	No	110 (26.1)
Materials	PFM	242 (57.5)
	Zirconia	165 (39.2)
	Other	14 (3.4)
Types	Crown	307 (72.9)
	3 units bridge	114 (27.1)
Gender	Male	184 (43.7)
	Female	237 (56.3)
Location	Anterior	142 (33.7)
	Posterior	279 (66.3)
Duration	< 6 months	33 (7.8)
	6 months – 1 year	56 (13.3)
	1-5 years	119 (28.3)
	5-10 years	134 (31.8)
	> 10 years	79 (18.8)

A chi-square test was employed to investigate the association between restorations and other factors, such as gender, location, and duration. The findings of the study revealed a statistically significant association (p < 0.05) between the characteristics mentioned earlier, as illustrated in Table 5.

**Table 5:** Correlation among various parameters recorded in the present study FPD: fixed dental prosthesis

Variables	Gender	Location	Duration
Technical findings			
Marginal integrity	0.004	0.055	0.118
Morphology	0.197	0.379	0.020
Color	0.447	0.456	0.001
Porcelain fracture	0.335	0.500	0.360
Biological findings			
Periodontal depth	0.015	0.026	0.000
Gingiva bleeding	0.124	0.000	0.000
Periapical lesion	0.203	0.241	0.000
Looseness of FPD	0.524	0.538	0.010
Dental carious	0.175	0.051	0.000
Other parameters			
Satisfaction	0.018	0.272	0.000
Materials	0.299	0.002	0.000
Types	0.119	0.052	0.213

## Discussion

FPD has long been accepted as the standard of care as a treatment option in dentistry for the replacement of one or more missing teeth. In addition, FPDs were long recognized as the most effective option for treating a single missing tooth [10].

Ha'il Dental Polyclinics Center practice performed more crowns and FPDs than primary healthcare centers. More crown and FPDs are employed by the National Health Service (NHS) in other nations, including the United Kingdom, than by private practices [11].

The findings presented in this study align with the previous studies, which indicated a higher failure risk for restorations exceeding a duration of five years [12-14]. Cheung et al. found a failure rate of 20.7% with an average service duration of 35 months [12]. Moreover, Libby et al. reported a failure rate of 15% with an average service period of 16 years, which translates to a mean lifespan of 6.3 years. In a 15-year prospective research, Vaulderhaug et al. found that failure rates were 4%, 12%, and 32% after five, 10, and 15 years, respectively [14].

Many crucial aspects, including speech and function, periodontal health, maxillomandibular relations, comfort, aesthetics, and ongoing assessment of the fixed prosthodontic treatment plan, were highlighted by researchers as being concerned with provisional restorations [15,16]. When all parties are aware of the goals and constraints of the treatment, provisional therapy can also be a useful tool in the psychological management of patients [17]. An acceptable length of time between tooth preparation and the finish of definitive treatment is necessary for the use of provisional restorations. Provisional treatment is typically well tolerated when this occurs. Extended usage times may increase tooth sensitivity and may cause pulp damage [18]. In the current study, poor color matching, unacceptable crown morphology, and faulty margins were recorded for crown-related problems. These results were in line with those of Goodacre et al., who noted that one of the most frequent issues with crowns was porcelain fractures and faulty margins [6]. Based on the current study, 22.8% of the cases needed endodontic treatment because of periapical lesions. This treatment might also involve the fabrication of new dental crowns. Goodacre et al. have highlighted that this is among the most frequent complications associated with single crowns that necessitate endodontic treatment. In certain instances, this might be partially explained by the very short average term of service in some cases. Porcelain fractures and defective margins affected both FPDs and crowns. The porcelain used intraorally is brittle. Fractures can be caused by high occlusal stresses, trauma, low elastic modulus of the metal alloy, incompatible coefficients of thermal expansion between the porcelain and the metal alloy, poor design, and micro-defects in the porcelain material [19]. This problem typically poses an aesthetic challenge because of the exposed underlying metal, particularly in the esthetic zone.

In the current study, the crowns and FPDs had defective margins that were irreparable (21.1%). This presents a considerably more serious issue that is sometimes unresolvable and requires prosthesis replacement [20]. The quality work among the clinicians who deliver these crowns based on their ranking may lead to defective margins such as inadequate gingiva retraction during impression-taking or poorly planned finish lines might lead to clinical mistakes. These mistakes could also be caused by air bubbles within the impression's margin. Over-contoured crowns, subgingival margin positioning, and inadequate marginal adaptation can all worsen localized periodontal inflammation. A longitudinal research was carried out by Vaulderhaug et al. to evaluate periodontal problems in patients with FPD [21]. They found that the gingiva index scored 2 and 3 with crowns more frequently observed.

Long-term success requires accurate marginal fit of indirect restorations. This is because ill-fitting margins make the tooth more susceptible to cement dissolution; once this occurs, marginal leakage occurs, which usually results in secondary caries and, if unnoticed, can lead to abutment vitality loss. Ill-fitting margins also cause plaque retention, predisposing the abutment to caries. Defective subgingival margins may jeopardize gingival health by altering local bacteria.

After a long period, biological complications such as caries and loss of vitality have been observed [22]. Some studies found no secondary caries [23-25]. On the other hand, Solá‐Ruíz et al. found that secondary caries was the main reason for biological complications after seven years of follow‐up [26]. Secondary caries was discovered in 10% of the FPDs in the current study. This could be an indication that patients chosen for these types of treatments were well-motivated patients who practiced good oral hygiene and thus had a low caries risk. On the other hand, the presence of defective margins in many of the failed restorations may imply that these crowned and abutment teeth will be susceptible to caries and loss of vitality in the long run.

PFM has long been regarded as the gold standard for prosthetic fabrication due to its ability to combine good mechanical properties with acceptable esthetic results, as well as its ability to provide the biological quality required for periodontal health [27]. This could explain why it was chosen for the majority (n=242; 57.5%) in this study.

Patients' perceptions of success have been shown to differ from those of clinicians. It was interesting to note that patient attitudes toward the prostheses did not always match the clinician's findings. Out of 421 prostheses, 311 (73.9%) were rated as satisfactory by patients. As a result, patient satisfaction may be a reliable predictor of success with their technical and biological findings. According to a study conducted by Agrawal et al., patients' willingness to accept fixed prostheses is based on various factors [28]. These include the dentist's treatment procedure, the patient's perception of the prosthesis' functional ability and appearance, and the patient's oral interpretation and discriminatory skill for the external contour of the prosthesis.

Porcelain that has broken can be repaired using a variety of techniques; these techniques usually entail attaching resin composite to the broken porcelain. The low color stability of resin composites, higher wear on the composite relative to porcelain, and the composite's deteriorating binding strength to porcelain with time all contribute to this technique's poor long-term prognosis [19]. Additionally, because porcelain and composite have different optical qualities, it is challenging to match colors, which often leads to less-than-ideal aesthetics.

The present study revealed that male patients had a more prominent open crown margin than female patients, which could be due to differences in oral hygiene maintenance. According to Yun-Han Ma et al., men tend to neglect their oral hygiene more than women [29]. They often fail to brush and dental floss, which can lead to a higher risk of plaque accumulation. This, in turn, may affect the marginal integrity of their teeth. The current study shows that most of the FDPs were inserted on posterior teeth rather than the anterior region. This is because clinical indication to place FDPs in the posterior area is more logical than in the anterior region. In a retrospective study conducted by Alenezi and Aloqayli, it was reported that long-span FDPs in the molar region can be associated with higher risks of failure compared to FDPs in the anterior region [30]. 

The current study represented clinical evaluation and periapical status of fixed prosthodontic restorations in a selected Saudi population, and it provided a theoretical basis for clinical care to some extent. The sample size and experimental method had a significant impact on the outcomes of fixed prosthodontic restorations. However, there are a few drawbacks that must be addressed. Because this was a single-center study, the sample size should have been larger. Furthermore, multicenter research using advanced techniques such as cone-beam computed tomography (CBCT) may be able to overcome the limitations of the current study. The outcome of the present study may be affected by various factors, such as different doctors rehabilitating patients, ridge form, saliva flow, mucosal quality, patient expectations, psychological profiles, and perceived aesthetics.

## Conclusions

Dental caries, periapical pathology, crown morphology, deficient margin, color mismatching, and periodontal disease are among the common issues that crowns and fixed restorations encounter. Some crown defects could be more effectively repaired rather than replaced. Most patients expressed satisfaction with the restoration. Consequently, patient satisfaction could be a good indicator of future success and survival rate. 
